# Associations between disturbed sleep and attenuated psychotic experiences in people at clinical high risk for psychosis

**DOI:** 10.1017/S0033291724000400

**Published:** 2024-07

**Authors:** M. J. C. Formica, M. Fuller-Tyszkiewicz, U. Reininghaus, M. Kempton, P. Delespaul, L. de Haan, B. Nelson, A. Mikocka-Walus, L. Olive, S. Ruhrmann, B. Rutten, A. Riecher-Rössler, G. Sachs, L. Valmaggia, M. van der Gaag, P. McGuire, J. van Os, EU-GEI High-Risk Study, J. A. Hartmann

**Affiliations:** 1 Orygen, Parkville, Australia; 2 Centre for Youth Mental Health, The University of Melbourne, Parkville, Australia; 3 Centre for Social and Early Emotional Development, School of Psychology, Deakin University, Geelong, Australia; 4 Department of Public Mental Health, Central Institute of Mental Health, Medical Faculty Mannheim, University of Heidelberg, Mannheim, Germany; 5 Department of Psychosis Studies, Institute of Psychiatry, Psychology, King's College London, London, UK; 6 Facalty of Health, Medicine and Life Sciences, Psychiatrie & Neuropsychologie, Maastricht University, Maastricht, The Netherlands; 7 Mondriaan Mental Health Centre, Maastricht/Heerlen, The Netherlands; 8 Department of Psychiatry, Early Psychosis, Academic Medical Centre, University of Amsterdam, Amsterdam, The Netherlands; 9 Institute for Mental and Physical Health and Clinical Translation, School of Medicine, Deakin University, Geelong, Australia; 10 Department of Psychiatry and Psychotherapy, Faculty of Medicine and University Hospital, University of Cologne, Cologne, Germany; 11 Department of Psychiatry and Neuropsychology, Faculty of Health, Medicine and Life Sciences, School for Mental Health and Neuroscience (MHeNS), European Graduate School of Neuroscience (EURON), Maastricht University Medical Centre, Maastricht, The Netherlands; 12 Medical Faculty, University of Basel, Basel, Switzerland; 13 Medical University of Vienna, Vienna, Austria; 14 Department of Psychology, Institute of Psychiatry, Psychology and Neuroscience, King's College London, London, UK; 15 Department of Clinical Psychology, University of Amsterdam, Amsterdam, The Netherlands; 16 Department of Psychiatry, University of Oxford, Warneford Hospital OX3 7JX, UK; 17 Department of Psychiatry, Utrecht University Medical Centre, Utrecht, The Netherlands

**Keywords:** etiology, early intervention, experience sampling methodology, psychotic symptoms, sleep disturbance

## Abstract

**Background:**

Pre-diagnostic stages of psychotic illnesses, including ‘clinical high risk’ (CHR), are marked by sleep disturbances. These sleep disturbances appear to represent a key aspect in the etiology and maintenance of psychotic disorders. We aimed to examine the relationship between self-reported sleep dysfunction and attenuated psychotic symptoms (APS) on a day-to-day basis.

**Methods:**

Seventy-six CHR young people completed the Experience Sampling Methodology (ESM) component of the European Union Gene-Environment Interaction Study, collected through PsyMate® devices, prompting sleep and symptom questionnaires 10 times daily for 6 days. Bayesian multilevel mixed linear regression analyses were performed on time-variant ESM data using the *brms* package in R. We investigated the day-to-day associations between sleep and psychotic experiences bidirectionally on an item level. Sleep items included sleep onset latency, fragmentation, and quality. Psychosis items assessed a range of perceptual, cognitive, and bizarre thought content common in the CHR population.

**Results:**

Two of the seven psychosis variables were unidirectionally predicted by previous night's number of awakenings: every unit increase in number of nightly awakenings predicted a 0.27 and 0.28 unit increase in feeling unreal or paranoid the next day, respectively. No other sleep variables credibly predicted next-day psychotic symptoms or vice-versa.

**Conclusion:**

In this study, the relationship between sleep disturbance and APS appears specific to the item in question. However, some APS, including perceptual disturbances, had low levels of endorsement amongst this sample. Nonetheless, these results provide evidence for a unidirectional relationship between sleep and some APS in this population.

## Background

Quality sleep is an essential feature of overall health and well-being with hypothesized roles in the maintenance of bodily systems, neural restoration, and cognitive functioning (Deak & Stickgold, 2010; Kyriacou & Hastings, 2010; Xie et al., 2013). Sleep dysfunction is a known and prevalent characteristic of multiple mental health disorders, and is particularly prominent among individuals with psychosis (Freeman, Sheaves, Waite, Harvey, & Harrison, 2020). Approximately 80% of patients with psychotic disorders report disturbed sleep (Bagautdinova et al., 2023; Cohrs, 2008; Davies, Haddock, Yung, Mulligan, & Kyle, 2017), with estimates of diagnosable sleep disorders ranging from 20% to 80% (Batalla-Martín et al., 2020; Reeve, Sheaves, & Freeman, 2019b), the most common of which are insomnia (i.e. the inability to fall or stay asleep), and nightmare disorder (i.e. recurrent episodes of waking following distressing dream content) (Batalla-Martín et al., 2020; Nuzum, Hammoud, Spencer, Akande, & Tognin, 2022; Reeve et al., 2019b). The presence of sleep disruptions for those across the psychosis continuum have been associated with widespread impacts on psychopathology, functioning, cognitive abilities, and has been found to be an important mediator for psychosis expression (Fekih-Romdhane et al., 2023; Freeman et al., 2017; Lunsford-Avery, Dean, & Mittal, 2017a; Lunsford-Avery et al., 2017b; Lunsford-Avery, LeBourgeois, Gupta, & Mittal, 2015; Manoach et al., 2014; Manoach & Stickgold, 2019).

A variety of research has indicated that profound subjective and objective abnormalities in sleep structure and quality have been observed prior to the onset of a threshold diagnosis in those at clinical high risk (CHR) for developing a psychotic disorder (Bagautdinova et al., 2023; Waite, Sheaves, Isham, Reeve, & Freeman, 2020; Zaks et al., 2022). The CHR criteria are predominately based on the presence of attenuated psychotic symptoms (APS), or brief duration frank psychotic symptoms, occurring in the context of a marked functional decline (Yung et al., 2005). Sleep disturbances parallel those seen in full-threshold psychosis populations and include circadian desynchrony, poor subjective sleep quality, and reduced quantity of sleep (Goines et al., 2019; Lunsford-Avery et al., 2017a; Lunsford-Avery et al., 2015; Poe et al., 2017; Reeve et al., 2019a). Reported sleep quality appears to be specifically related to attenuated symptom domains of paranoia and perceptual abnormalities (Goines et al., 2019; Kasanova, Hajduk, Thewissen, & Myin-Germeys, 2020).

The presence of such associations has led researchers to suspect that sleep disturbance has an important mechanistic role in the pathophysiology of psychotic disorders (Bagautdinova et al., 2023). Experimental and interventional research supports this idea of sleep disturbances being potentially causal in the expression of psychotic symptoms (Bradley et al., 2018; Freeman et al., 2017; Reeve, Emsley, Sheaves, & Freeman, 2018; Waite et al., 2023). A large single-blind randomized control trial (RCT) of university students experiencing insomnia (*n* = 3375) indicated that a digital intervention for insomnia was associated with consistent improvements over 22 weeks in sleep disturbance, paranoia, and hallucinations (Freeman et al., 2017). The improvement in paranoia and hallucination symptoms has been supported in those meeting insomnia criteria in the CHR population, with a case-series (Bradley et al., 2018) and a recent RCT of 40 participants (Waite et al., 2023) showing preliminary support for up to a 9-month symptomatic improvement following the reception of tailored sleep intervention.

Despite this, longitudinal investigations into the sleep–psychosis relationship have revealed conflicting levels of support, with some studies reporting no direct relationship between sleep disturbance and the later development of threshold psychotic disorder once accounting for previous symptom severity (Formica et al., 2023; Nuzum et al., 2022; Reeve et al., 2019a) and others reporting longitudinal relationships of varying strengths (Haidl et al., 2018; Lunsford-Avery et al., 2017b; Lunsford-Avery et al., 2015; Ruhrmann et al., 2010; Wang et al., 2023; Zaks et al., 2022; Zhou et al., 2022). The variation in follow-up time periods, as well as differences in the aspects of both sleep disturbance and APS under assessment, may contribute to some of these discrepancies. This has led to a greater focus on utilizing Experience Sampling Methodology (ESM) approaches to understand how sleep may maintain or exacerbate APS in a highly nuanced manner on a shorter term or day-to-day basis. Theoretical models and ESM research has hypothesized that the sleep–psychosis relationship is bidirectional (Blanchard, Andrea, Orth, Savage, & Bennett, 2020; Boland, Gallagher, & Clarke, 2022; Reeve et al., 2019a; Rehman et al., 2017). Specifically, it is suggested that poor sleep can lead to a greater propensity for hallucinations, delusions, and thought disorder, while these psychotic symptoms are reciprocally associated with difficulties sleeping (Boland et al., 2022; Waite et al., 2018). It has also been proposed that the reduction of social and occupational functioning and reduced daily activity common in psychotic disorders can disrupt circadian rhythmicity and prompt further sleep disruptions and vice versa (Harvey, Murray, Chandler, & Soehner, 2011; Waite et al., 2016). The existing ESM research has predominately examined the sleep–psychotic symptom relationship in psychotic and community samples (Hennig & Lincoln, 2018; Kasanova et al., 2020; Meyer et al., 2022), and largely supports the notion of bidirectionality. Equivalent investigation within CHR populations has been limited, with only one study completed to date. van der Tuin et al. (2023) showed within-person effects of poor sleep predicting greater expression of psychotic symptoms, and a weaker, between-person bidirectional relationship between the symptoms. This analysis, however, lacked investigation into the specific psychotic symptoms that were exacerbated by sleep disturbance, which is essential for building a nuanced understanding of the specific effects that sleep disturbance may have on psychotic experiences. Thus, further investigation into the directionality and specificity of a sleep–psychotic symptoms relationship in CHR samples is warranted.

This study aims to address the current literature gaps through investigating the temporal dynamics on a day-to-day basis between self-reported sleep disturbance and attenuated psychotic experiences in a CHR sample. An additional exploratory aim is to investigate the 6-month longitudinal relationship between sleep disturbance and APS. Through exploring symptom-specific bidirectionality of temporal relationships in a CHR sample, we hope to elucidate whether self-reported sleep disturbance may be an important preceding or maintaining factor for the development of psychotic symptoms. We hypothesized that there is a bidirectional day-to-day relationship between self-reported sleep and APS in CHR patients. Hence, we hypothesize that previous night's sleep will predict higher levels of next day attenuated psychotic experiences and vice versa. There are no specific hypotheses surrounding the specificity of these relationships, with an exploratory approach being taken.

## Method

### Participants

Participants were recruited through the European Network of National Schizophrenia Networks studying Gene-Environment Interactions (EU-GEI) High Risk Study which sought to prospectively identify factors that predicted adverse clinical outcomes in people at CHR for psychosis (European Network of National Networks studying Gene-Environment Interactions, 2014). Recruitment occurred between 2010 and 2015 within clinical settings of several countries across Europe, Asia, and Australia. Each site gained ethics approval from local bodies prior to recruitment. The overall sample of the EU-GEI High Risk Study comprises 411 young people. All participants completed a baseline visit and were then invited for follow-up visits at 6, 12, and 24 months later. Typical age of participants was 18–35 years but not restrictive due to variation between sites in the age at which persons are accepted by clinical services. Participants were included if they provided informed consent, and met CHR criteria according to the Comprehensive Assessment of At-Risk Mental States (CAARMS) (Yung et al., 2005). Participants with an IQ less than 60 or any documented history of developmental delays were excluded from participating. The ESM sample comprises of those who opted-in to this study component resulting in 79 CHR individuals from the overall EUGEI sample. These participants were recruited from study centers in Amsterdam, The Hague, London, and Melbourne. The data from this ESM sample are used in this research.

### Measures

#### ESM

ESM consists of assessing the participant's experiences in the flow of daily life (e.g. stressful life events, reactions to events, participant's context) rather than via research interview or questionnaire. Data were gathered through a mobile device (called a PsyMate®, see www.psymate.eu) that was held by the participant for 6 days. The device was programmed to emit beeps signaling a survey 10 times per day at random moments within 90 min time blocks. The participant is asked to respond immediately, but not later than 15 min to a list of items. Items selected for use have been used widely amongst previous studies, for example, see Reininghaus et al. (2019), Delespaul (1995), Wigman et al. (2013). A full list of the ESM questions and response options is included in Table 1.

**Table 1.** ESM questions and response options for each construct of interest

Construct	Question	Answer options
Sleep	How long did it take before I fell asleep last night	0 = 0–5 min, 1 = 5–15 min, 2 = 15–30 min, 3 = 30–45 min, 4 = 45 m–1 h, 5 = 1–2 h, 6 = 2–4 h, 7 = >4 h
	How often did I wake up last night	1 = 0, 2 = 1, 3 = 2, 4 = 3, 5 = 4, 6 = 5, 7 = >5
	How long did I lie awake this morning before getting up	0 = 0–5 min, 1 = 5–15 min, 2 = 15–30 min, 3 = 30–45 min, 4 = 45 m–1 h, 5 = 1–2 h, 6 = 2–4 h, 7 = >4 h
	I slept well	7-point scale, 1 = no, 7 = very
Positive affect	I feel cheerful	7-point scale, 1 = no, 7 = very
	I feel relaxed	7-point scale, 1 = no, 7 = very
	I feel enthusiastic	7-point scale, 1 = no, 7 = very
	I feel satisfied	7-point scale, 1 = no, 7 = very
Negative affect	I feel down	7-point scale, 1 = no, 7 = very
	I feel lonely	7-point scale, 1 = no, 7 = very
	I feel anxious	7-point scale, 1 = no, 7 = very
	I feel insecure	7-point scale, 1 = no, 7 = very
	I feel annoyed	7-point scale, 1 = no, 7 = very
Attenuated psychotic experiences	It's hard to express my thoughts in words	7-point scale, 1 = no, 7 = very
	My thoughts are influenced by others	7-point scale, 1 = no, 7 = very
	I can't get my thoughts out of my head	7-point scale, 1 = no, 7 = very
	I feel unreal	7-point scale, 1 = no, 7 = very
	I feel paranoid	7-point scale, 1 = no, 7 = very
	I hear things that aren't really there	7-point scale, 1 = no, 7 = very
	I see things that aren't really there	7-point scale, 1 = no, 7 = very

#### Psychopathology

*Comprehensive Assessment of At-Risk Mental States (CAARMS)*. The CAARMS is a semi-structured interview that is designed to identify CHR individuals with its assessment of APS and social functioning (Yung et al., 2005). CAARMS APS comprises four domains, each with an intensity and frequency rating from 0 to 6, the distress experienced due to the symptom from 0 to 100, and the pattern of symptoms with substance use from 0 to 2. These domains are Unusual Thought Content, comprising bizarre thought content, including ideas of reference, and thought insertion; Non-Bizarre Ideas, comprising non-bizarre thought content such as persecutory, jealous, or guilt-laden beliefs; Perceptual Abnormalities, including perceptual distortions and hallucinatory content; and Disorganized Speech. The supplementary analysis investigated the domains comprising APS separately to determine the distinct relationships with self-reported sleep disturbance. Continuous measures of each domain have been used, created as per previous research through multiplying the symptom intensity by its frequency rating with domain scores reflecting this product of frequency and intensity ratings (ranging from 0 to 36). Higher scores indicate a greater symptom severity (Morrison et al., 2012; Wilson, Yung, & Morrison, 2020).

*Twenty-four-item Brief Psychiatric Rating Scale (BPRS)*. The 24-item BPRS is a measure used to assess psychopathology in individuals with psychotic illnesses, with each item scored on a scale from one to seven (Ventura et al., 1993). This measure is psychometrically sound, with the ability to garner clinically meaningfully interpretations (Leucht et al., 2005). The BPRS has a four factor structure, comprising domains of negative symptoms, positive symptoms, depression/anxiety symptoms, and hostility/uncooperativeness (Ventura, Nuechterlein, Subotnik, Gutkind, & Gilbert, 2000). The sum score was used within the supplementary analyses, with a range of possible values from 24 to 168.

### Data analysis

All data cleaning, preparation, and analyses were completed using R version 4.3.0 and RStan version 2.26.1. Participants with less than 33% response rate to ESM prompts were excluded from analyses after ensuring that response rate was not associated with each of the variables of interest or relevant demographic variables. This response criterion has been widely used as a guideline for minimum ESM completion required for analysis (Delespaul, deVries, & van Os, 2002; Palmier-Claus et al., 2011; Reininghaus et al., 2019). ESM data display a multi-level structure where repeated measurements of variables (beeps) are nested within each day, and further nested within the level of the individual. Within this analysis, day-level psychopathological variables were aggregated to day-level summaries; thus, two-level models were run with level 1 being day level and level 2 being the participant level. Descriptive statistics were generated on the level of the participant. Scatterplots and histograms were inspected prior to testing. The psychosis ESM variables were log-transformed due to displaying a positive skew (high level of lower responses), and daily group mean centered values were used as predictors in model estimation to disambiguate within- from between-individual effects (Enders & Tofighi, 2007). Intra-class correlations of ESM psychosis and sleep variables ranged between 0.64 and 0.79.

A series of Bayesian multilevel mixed linear regression analyses were performed on time-variant ESM data using the *brms* package to better address potential difficulties with power of the analysis (Lee & Song, 2004; van de Schoot et al., 2014). In Bayesian analysis, there is less emphasis on traditional power analysis and strict *p*-level adjustments for multiple testing – this is due to a focus on estimating effect sizes and their credible intervals, which provide a range of plausible values for the true effects (Gelman, 2014). Additionally, Bayesian analysis is not conducted under the assumption of the null hypothesis, instead representing a statistical summary of probability using posterior probability distributions (Sweet, 2016). Thus, there are no *p*-values reported within the results. Bayesian methods can yield informative posterior distributions which allow inference into the probability that a parameter falls within a range of values, rather than relying on arbitrary significance thresholds (Gelman, 2014). These assumptions underlying Bayesian analysis methods differ from typical frequentist approaches, and do not result in the same issue of assuming an ‘intersection null hypothesis’ which necessitates familywise-corrections (Berry & Hochberg, 1999). The default weakly informative priors of the *brms* package were set due to the paucity of relevant existing literature in prodromal illness states. The models were run with independent variables time-lagged for each model and fixed effects for the beep order and lagged time included. The day-to-day associations between sleep and psychotic experiences were investigated with, (i) previous night's sleep as predictor and subsequent daytime psychotic experiences as outcome, and (ii) daytime psychotic experiences as predictor and subsequent sleep as outcome. For any meaningful effects, negative affect, psychotropic medication, nicotine, cannabis, and alcohol use were then included in the model to determine if they accounted for the observed predictive effects. All models were run with 2000 warm-up iterations followed by 4000 post-warm-up draws and two chains. The exploratory longitudinal supplementary analyses were then investigated through modelling the effects of self-reported sleep (both its mean and intraindividual variability [IIV]) at baseline predicting 6-month follow-up APS, controlling for baseline APS. The IIV was calculated through the standard deviation approach using the *varian* package. Only the 6-month data were used in the supplementary analysis due to the significant attrition observed at subsequent follow-up timepoints. All generated models converged and illustrated R-hat values of one, posterior predictive plots were also inspected and showed good model fit.

## Results

Table 2 displays sample characteristics at baseline. Each participant completed questions from a mean average of 38.74 surveys (65% compliance, s.d. = 10.29, median = 37.00). A total of 76 of the 79 individuals’ data met compliance criteria specified previously. Descriptive statistics for longitudinal analysis variables can be seen in online Supplementary Table S1.

**Table 2.** Demographic and ESM descriptive statistics of sample

		M (s.d.) *n* = 76
Age		23.07 (5.00)
Sex		35 males; 41 females
Recruitment site	*London*	36
	*Amsterdam*	3
	*The Hague*	23
	*Melbourne*	14
*Positive affect*		3.45 (1.30)
*Negative affect*		3.19 (1.41)
Attenuated psychotic experiences	It's hard to express my thoughts in words	3.00 (1.68)
	My thoughts are influenced by others	2.30 (1.46)
	I can't get my thoughts out of my head	3.04 (1.71)
	I feel unreal	2.37 (1.62)
	I feel paranoid	2.45 (1.68)
	I hear things that aren't really there	1.78 (1.33)
	I see things that aren't really there	1.54 (1.03)
Sleep	How long did it take before I fell asleep last night	2.44 (1.62)
	How often did I wake up last night	2.00 (1.63)
	How long did I lie awake this morning before getting up	2.21 (1.43)
	I slept well	3.98 (1.49)

### Reported sleep disturbances predicting next day attenuated psychotic symptoms

Summary statistics for the analyses considering the predictive effects of previous night's sleep on next day psychotic symptoms can be seen in Table 3. With 95% confidence, two of the seven symptom measures were predicted by the previous night's number of awakenings. Specifically, every unit increase in number of awakenings predicted a 0.27 and 0.28 unit increase in feeling unreal or paranoid the next day, respectively. In real terms, this indicates that each subjective awakening during the night will be associated with a small increase in feeling unreal or paranoid the next day. These relationships were not affected by adding previous day negative affect, psychotropic medication, nicotine, alcohol, or cannabis use as covariates. However, next-day paranoia was additionally predicted by cannabis use (M = 1.18, 95% CI 0.001–2.37), alcohol use (M = −2.51, 95% CI −4.23 to −0.78), and negative affect (M = 0.25, 95% CI 0.07–0.42). No other sleep variables meaningfully predicted changes in next-day psychotic symptoms.

**Table 3.** Model estimates for previous night's sleep predicting next day psychotic symptoms

Predictor	It's hard to express my thoughts in words	My thoughts are influenced by others	I can't get my thoughts out of my head	I feel unreal	I feel paranoid	I hear things that aren't really there	I see things that aren't really there
	M [95% CI]	M [95% CI]	M [95% CI]	M [95% CI]	M [95% CI]	M [95% CI]	M [95% CI]
Time taken to fall asleep	0.02 [−0.17, 0.21]	0.08 [−0.05, 0.21]	0.07 [−0.12, 0.25]	0.06 [−0.08, 0.22]	0.05 [−0.10, 0.21]	−0.06 [−0.17, 0.05]	0.06 [−0.05, 0.18]
Number of awakenings during the night	0.13 [−0.13, 0.39]	0.11 [−0.07, 0.29]	0.11 [−0.15, 0.36]	0.28 [0.05, 0.50]*	0.27 [0.03, 0.51]*	0.11 [−0.05, 0.27]	0.09 [−0.08, 0.26]
Time awake in bed prior to rising	0.04 [−0.12, 0.19]	−0.06 [−0.16, 0.05]	−0.03 [−0.18, 0.12]	0.05 [−0.09, 0.18]	−0.02 [−0.15, 0.11]	−0.04 [−0.13, 0.05]	0.00 [−0.09, 0.10]
Subjective sleep quality	0.14 [−0.09, 0.38]	0.09 [−0.07, 0.26]	0.05 [−0.02, 0.28]	0.09 [−0.11, 0.29]	0.04 [−0.16, 0.24]	0.01 [−0.13, 0.15]	0.13 [−0.01, 0.29]

Estimates provided refer to the means and 95% credible intervals of the posterior distributions.

*95% Credible interval does not include a 0 value.

### Attenuated psychotic symptoms predicting self-reported sleep disturbance

Summary statistics for the analyses considering the predictive effects of daytime psychotic symptoms on next reported sleep quality can be seen in Table 4. No psychotic symptom credibly predicted a change in next day reported sleep quality.

**Table 4.** Model estimates for psychotic symptoms predicting next day sleep disturbance

	Time taken to fall asleep	Number of awakenings during the night	Time awake in bed prior to rising	Subjective sleep quality
Predictor	M [95% CI]	M [95% CI]	M [95% CI]	M [95% CI]
It's hard to express my thoughts in words	0.38 [−0.17, 0.95]	0.24 [−0.24, 0.71]	0.04 [−0.50, 0.58]	−0.18 [−0.65, 0.29]
My thoughts are influenced by others	−0.26 [−1.02, 0.50]	−0.07 [−0.70, 0.57]	0.39 [−0.34, 1.10]	−0.12 [−0.77, 0.51]
I can't get my thoughts out of my head	−0.21 [−0.94, 0.51]	0.15 [−0.49, 0.89]	−0.54 [−1.24, 0.15]	−0.14 [−0.76, 048]
I feel unreal	−0.31 [−0.92, 0.51]	−0.38 [−0.88, 0.13]	−0.36 [−0.96, 0.21]	0.43 [−0.04, 0.92]
I feel paranoid	−0.09 [−0.88, 0.71]	0.10 [−0.57, 0.78]	0.42 [−0.38, 1.20]	−0.21 [−0.89, 0.44]
I hear things that aren't really there	−0.01 [−0.77, 0.77]	−0.25 [−0.91, 0.41]	0.12 [−0.60, 0.85]	0.23 [−0.42, 0.86]
I see things that aren't really there	0.31 [−0.53, 1.12]	0.03 [−0.66, 0.74]	−0.32 [−1.17, 0.50]	−0.52 [−1.18, 0.16]

Estimates provided refer to the means and 95% credible intervals of the posterior distributions.

## Discussion

This study aimed to better understand the day-to-day relationship between self-reported sleep disturbance and APS in a CHR sample. Overall, the hypothesis that there would be a bidirectional day-to-day relationship between APS and sleep disturbance was not supported. The results indicate that the credible day-to-day relationships between these constructs were *unidirectional*, with the reported number of awakenings during sleep predicting next-day reports of feeling paranoid and unreal. There were no credible day-to-day relationships in the inverse direction.

### Day-to-day relationship between reported sleep disturbance and psychotic symptoms

Our finding that the reported number of awakenings predicts increases in next-day feelings of paranoia and being unreal even after controlling for negative affect and substance use is consistent with some previous findings conducted within other populations (Hennig & Lincoln, 2018; Hennig, Schlier, & Lincoln, 2020; Kasanova et al., 2020). The only previous CHR study considering the day-to-day relationship between sleep and psychosis symptom expression also found unidirectional day-to-day relationships (van der Tuin et al., 2023). Notably, in this study, specific psychotic symptom domains were not analyzed, and instead, a summed composite score over a period of 90 days of sleep and attenuated psychotic experience was used. Experimental sleep deprivation research additionally supports the short-term effect of disturbed sleep on the emergence of psychotic-like experiences, particularly paranoia (Kahn-Greene, Killgore, Kamimori, Balkin, & Killgore, 2007; Reeve et al., 2018). The importance of sleep loss in the expression of psychotic-like experiences is highlighted through self-reported number of awakenings being the only meaningful sleep construct predicting the experience of APS as this item reflects the closest surrogate marker of sleep deprivation assessed within this study.

Paranoia has received more research attention than feelings of unreality regarding exacerbation following sleep disturbance. Here, consistent associations are found, albeit inconsistent regarding the relevant aspect of sleep disturbance and directness of the effect (Hennig & Lincoln, 2018; Hennig et al., 2020; Kammerer, Mehl, Ludwig, & Lincoln, 2021; Kasanova et al., 2020; Meyer et al., 2022). Kasanova et al.'s (2020) results support our unidirectional finding, however, illustrated that the full effect of general subjective sleep disturbance on subsequent paranoia was mediated by negative affect. Moreover, Kammerer et al. (2021) illustrated in their psychosis sample with persistent delusions that only a unidirectional relationship between sleep disturbance predicting later persecutory delusions was present. This was specific to objective measures of sleep disturbance, namely, circadian disruption and sleep efficiency, with no associations found within subjective sleep reports. Conversely, Hennig and Lincoln (2018) found that lower subjective total sleep time and higher subjective dream frequency (both of which were not assessed within our current study) unidirectionally predicted the experience of next morning paranoia. Within interventional research, a unidirectional effect of improvements in insomnia symptoms leading to reduced paranoia is similarly found (Waite et al., 2023). Hence, sleep disturbance consistently predicts high levels of paranoia albeit with variations within and between modalities of sleep disturbance measurements. These findings globally align with a cognitive model of persecutory delusions which postulates that sleep disruptions are one key developmental and maintenance facet which serve to strengthen core threat beliefs through increasing mood dysregulation and the propensity to anomalous experiences (Freeman, 2016).

Temporal investigations of feelings of unreality being predicted by number of awakenings in a CHR sample are novel. The anomalous experience of ‘reality’, however, has been increasingly explored as relevant to the etiology of schizophrenia spectrum illnesses in recent years, with a focus on ‘self-disturbance’ being a trait marker of psychosis risk (Delf & Beattie, 2022; Drori, Bar-Tal, Stern, Zvilichovsky, & Salomon, 2020; Krcmar et al., 2024; Nelson et al., 2020). Being unable to trust or clearly differentiate real from imagined experience is essential for navigating day-to-day life, strongly overlaps with dissociative phenomena, and has been stipulated as a risk for the experience of later hallucinations due to associations with source monitoring deficits (Černis, Freeman, & Ehlers, 2020; Fazekas, 2020; Longden et al., 2020; Waters et al., 2012). Previous associations between insomnia and dissociative experiences have been illustrated in both cross-sectional literature and some experimental research within healthy populations (Barton, Kyle, Varese, Jones, & Haddock, 2018; van Heugten – van der Kloet, Giesbrecht, & Merckelbach, 2015). This association may be explained by difficulties in distinguishing between states of consciousness that are worsened following fragmented sleep. Though purely speculative and requiring direct research focus, some of this relationship may be related to the high prevalence of nightmares and nightmare disorder found within CHR samples (Reeve et al., 2019b). Having intense dreams which result in a high threat response and may also linger into experiences in regular consciousness and bias thinking points to issues with aberrant salience and sensory hyperarousal which may lead to psychotic symptom expression, including feelings of unreality or dissociation on a day-to-day basis (Sheaves, Rek, & Freeman, 2023; Wamsley, Donjacour, Scammell, Lammers, & Stickgold, 2014; Waters et al., 2012). This potential mechanistic pathway has been speculated within the narcolepsy research (Hanin et al., 2021), as well as within a recent review of nightmares effects of psychopathology transdiagnostically (Sheaves et al., 2023). The experience of nightmares within CHR populations and the relationship with symptoms on a day-to-day basis may thus be an area for future early psychosis research focus.

The absence of a day-to-day relationship between reported sleep disturbance and hallucination items contrasts with the existing research across study designs (Hennig et al., 2020; Petrovsky et al., 2014; Reeve et al., 2018; Waters, Chiu, Atkinson, & Blom, 2018). Within the ESM literature, Hennig et al. (2020) found a significant interaction between both subjective and objective sleep disturbance predicting next day hallucinatory experiences by the level of psychosis proneness of an individual; this relationship was not present for paranoia. The more comprehensive measurement of hallucinatory experiences in the Hennig study compared with our own may contribute to some of these differences, particularly given the low level of endorsement of these items in this study. However, as informed by sleep deprivation studies, it may instead be that the most consistent predictor for perceptual abnormalities is the degree of sleep loss (Waters et al., 2018), of which a direct measurement was not included within this study. Thus, it may be that the absence of this measurement has resulted in this conflicting result. Moreover, the sample within this project exhibited only mild-to-moderate levels of sleep disturbance, and hence, potentially did not experience the level of sleep disturbance necessary to elicit perceptual disturbances. This postulation is supported when considering studies which show improvements in hallucination severity following sleep interventions for those with diagnosable sleep disorders (Bradley et al., 2018; Freeman et al., 2017; Waite et al., 2023)

### Implications

The findings of reported sleep fragmentation leading to the expression of next-day feelings of unreality and paranoia support the notion that sleep disturbance may not purely act as a by-product of psychotic symptomatology and may indeed have an active role in the expression and maintenance of some psychotic symptoms in daily life. This indicates that sleep fragmentation may be a potential target for intervention for those experiencing paranoia and feelings of unreality. The presence of these associations within a CHR sample additionally supports the notion that the impacts of sleep on psychotic symptoms occur across the illness continuum and can be investigated and potentially intervened prior to the onset of full-threshold disorders. The specificity of this to paranoia and feeling unreal may indicate that these symptoms are uniquely impacted by experienced disruptions in sleep continuity which can add to existing theoretical models of symptoms expression. Together, these findings support the importance of precision psychiatry, and the need to deliver treatments based on the specific pattern of symptoms and not by their overarching diagnostic label (Coutts, Koutsouleris, & McGuire, 2023).

### Strengths and limitations

While providing novel contributions to the literature, a number of limitations should be noted. This project only contained limited subjective measures of sleep disturbance, and these were only collected from a subset of total EU-GEI CHR sample leading to low power for the supplementary longitudinal analysis. Moreover, although the sleep disturbance questions were selected due to their ability to be practically implemented in clinical practice, they do not provide nuanced information regarding either the objective or qualitative experience of sleep. For instance, including questions regarding nightmare presence or content of dreams may assist in building our understanding of psychosis phenomenology given the qualitative experience of those with psychotic illnesses experiencing difficulties in their ability to feel a sense of ‘mineness’ over their conscious experience (Nelson et al., 2020; Parnas et al., 2005; Waite et al., 2018; Waite et al., 2016). Similarly related to the measurement of sleep disturbance, an estimate of total sleep time and hence sleep deprivation, was lacking. The closest proxy measure of sleep deprivation was that of sleep fragmentation which was implicated as a predictor of later APS. Considering sleep deprivation, and consistency, of sleep loss may result in important associations with psychotic-like experiences and is an area for future research to investigate. Moreover, it is important to note that while we can make meaningful temporal inferences from the findings of this study, it is still correlational in nature and thus the potential causality of associations cannot be inferred.

This study has highlighted a larger issue within the CHR space regarding a paucity of optimal state measures for measuring APS. Although the ESM items included in this analysis have been used extensively by previous projects (Chun, Gross, Mielock, & Kwapil, 2020; Delespaul, 1995; Lardinois, Lataster, Mengelers, Van Os, & Myin-Germeys, 2011; Myin-Germeys, Marcelis, Krabbendam, Delespaul, & van Os, 2005; Paetzold et al., 2021; Wigman et al., 2013) most ESM psychosis items are adapted from research within full-threshold samples and thus may not reflect complete representations of the participants subjective experiences within pre-diagnostic states. For example, the prompt ‘I see things that aren't really there’ or even use of the word ‘paranoid’ may not elicit the participant to consider the array of attenuated disturbances which are elucidated in typical interviewer-assessed measures of the CHR state. This may be particularly relevant for attenuated perceptual alterations, which has low levels of endorsement across this sample despite Perceptual Abnormalities being at a comparable mean score to the Unusual Thought Content and Non-Bizarre Ideas domains of the CAARMS. An investigation of the relevance of ESM psychosis prompts amongst the CHR population specifically in future research may improve the applicability of questions. Finally, while compliance was moderate within this sample, the future use of smartphone-based approaches and passive assessment would promote a rich and minimally burdensome data collection procedure (Adler et al., 2020; Cella et al., 2019). Passive sensing has been found to improve rates of adherence and provide more comprehensive data in psychotic populations (Aledavood et al., 2019; Staples et al., 2017), though is not without its limitations given the low activity commonly seen in such populations.

## Conclusions

This study is one of the first to consider the specific day-to-day relationships between subjective sleep disturbance and APS in an international CHR sample. We found support for a unidirectional effect of sleep fragmentation predicting increases in reports of next-day paranoia and unreality. The specificity of relationships highlights the need for symptom-level approaches in understanding disorder etiology, and the need for precision psychiatry approaches for determining relevant interventions. The current Accelerating Medicines Partnership® program – Schizophrenia may present an opportunity to apply these symptom-level research approaches. Future research should improve daily assessment of sleep disorders within CHR populations, with a particular focus on the often-overlooked experience of nightmares and how this may contribute to symptom fluctuations. By continuing this exploration into modifiable risk factors this work may aid in the broader goal of psychosis prevention.

## Supporting information

Formica et al. supplementary material Formica et al. supplementary material

## Supplementary material

The supplementary material for this article can be found at https://doi.org/10.1017/S0033291724000400.
