# Supplementary material for: Associations between disturbed sleep and attenuated psychotic experiences in people at clinical high risk for psychosis
Source: Psychol Med. 2024 Mar 7;54(9):2254–63. doi: 10.1017/S0033291724000400 (PMC11413346; doi:10.1017/S0033291724000400)
Supplement: Formica et al. supplementary material [file S0033291724000400sup001.docx]

Supplementary Materials

A logistic binomial regression was run to assess whether participant drop out at Month 6 was predicted by any sample characteristics. Females were significantly less likely than males to be non-completers of the follow-up measure (β = -1.24, SE = 0.63, p = 0.048.

Table S1. Descriptive statistics for longitudinal analysis

|  | | Baseline (T1)  M (SD)  n=76 | Month 6 (T2)  M (SD)  n=54 |
| --- | --- | --- | --- |
| Age | | 23.07 (5.00) | 23.31 (5.25) |
| Sex | | 35 Males; 41 Females | 22 Males; 32 Females |
| Recruitment site | *London* | 36 | 25 |
|  | *Amsterdam* | 3 | 2 |
|  | *The Hague* | 23 | 19 |
|  | *Melbourne* | 14 | 8 |
| Unusual Thought Content | | 10.46 (8.56) | 7.95 (7.43) |
| Non-Bizarre Ideas | | 11.64 (9.84) | 9.15 (8.21) |
| Perceptual Abnormalities | | 10.01 (6.57) | 10.04 (7.72) |
| Disorganised Speech | | 5.59 (5.57) | 4.32 (5.67) |
| BPRS total score | | 44.33 (9.07) | 41.55 (10.44) |

**Longitudinal associations**

Summary statistics for the analyses considering the predictive effects of reported sleep in the ESM phase on 6-month follow-up psychotic symptoms can be seen in Table S5. Table S5 illustrates the model estimates of the posterior distribution calculated using both the IIV and mean of reported sleep items during the ESM phase. The only credible relationship highlighted was mean time spent in bed prior to rising negatively predicting follow-up unusual thought content. No other ESM sleep variable meaningfully predicted the level of attenuated psychotic symptom expression at 6-month follow-up.

Table S2. Model estimates for ESM sleep disturbance variables IIV and mean estimates predicting month 6 follow-up psychotic psychopathology. Estimates provided refer to the means and 95% credible intervals of the posterior distributions.

|  | UTC | NBI | PA | DS | BPRS |
| --- | --- | --- | --- | --- | --- |
| ***IIV Estimate Predictor*** | *M [95% CI]* | *M [95% CI]* | *M [95% CI]* | *M [95% CI]* | *M [95% CI]* |
| Time taken to fall asleep | -0.59 [-4.73, 3.65] | -0.62 [-5.53, 4.21] | -1.41 [-4.88, 1.96] | -2.55 [-7.10, 2.00] | 0.18 [-7.68, 8.41] |
| Number of awakenings during the night | -0.15 [-4.96, 4.70] | -0.67 [-6.38, 4.93] | 0.99 [-3.40, 5.47] | -0.07 [-4.15, 3.74] | 1.97 [-6.44, 10.39] |
| Time awake in bed prior to rising | -2.55 [-7.25, 2.08] | -3.89 [-9.84, 2.23] | -2.03 [-5.70, 1.52] | 2.50 [-1.47, 6.39] | -3.25 [-11.43, 5.14] |
| Subjective sleep quality | -0.65 [-4.95, 3.66] | -0.72 [-6.28, 4.87] | -1.91 [-5.21, 1.39] | -0.17 [-3.75, 3.51] | -1.15 [-8.28, 5.76] |
| ***Mean Estimate Predictor*** |  |  |  |  |  |
| Time taken to fall asleep | -0.27 [-2.58, 2.06] | -0.66 [-3.56, 2.37] | -0.65 [-2.47, 1.21] | 0.60 [-1.42, 2.65] | -1.18 [-6.05, 3.92] |
| Number of awakenings during the night | 0.58 [-1.15, 2.35] | -0.55 [-3.32, 2.19] | -0.34 [-1.80, 1.04] | -1.14 [-2.69, 0.43] | -0.64 [-4.47, 3.15] |
| Time awake in bed prior to rising | -2.12 [-4.15, -0.03]* | -1.42 [-3.91, 1.08] | -0.83 [-2.37, 1.56] | 0.69 [-0.96, 2.37] | 3.21 [-1.26, 7.95] |
| Subjective sleep quality | 1.00 [-1.24, 3.26] | -2.61 [-6.04, 0.75] | -0.39 [-2.37, 1.56] | 0.10 [-1.92, 2.08] | -1.55 [-5.82, 2.73] |

Note: UTC = Unusual Thought Content, NBI = Non Bizarre Ideas, PA = Perceptual Abnormalities, DS = Disorganised Speech , BPRS = Brief Psychiatric Rating Scale; *95% Credible Interval does not include a 0 value
